# Development and Validation of a Nomogram to Predict Cancer-Specific Survival for Middle-Aged Patients With Early-Stage Hepatocellular Carcinoma

**DOI:** 10.3389/fpubh.2022.848716

**Published:** 2022-02-28

**Authors:** Chong Wen, Jie Tang, Hao Luo

**Affiliations:** ^1^General Surgery Center, The General Hospital of Western Theater, Chengdu, China; ^2^College of Medicine, Southwest Jiaotong University, Chengdu, China; ^3^Department of Biostatistics and Epidemiology, School of Public Health, Shenyang Medical College, Shenyang, China

**Keywords:** nomogram, cancer-specific survival, middle-aged patients, hepatocellular carcinoma, SEER

## Abstract

**Background:**

Hepatocellular carcinoma is a common cause of death in middle-aged patients. We aimed to construct a new nomogram to predict cancer-specific survival (CSS) in middle-aged patients with hepatocellular carcinoma at an early stage.

**Method:**

We collected clinicopathological information on early middle-aged patients with hepatocellular carcinoma from the SEER database. Univariate and multivariate Cox regression models were used to screen the independent risk factors for prognosis. These risk factors were used to construct predictions of CSS in patients with hepatocellular carcinoma. Consistency index (C- index), calibration curve, area under the receiver operating curve (AUC) were used. A decision analysis curve (DCA) was used to evaluate the clinical utility of the predictive model.

**Results:**

A total of 6,286 patients with hepatocellular carcinoma in early middle age were enrolled. Univariate and multivariate Cox regression analysis showed that sex, marriage, race, histological tumor grade, T stage, surgery, chemotherapy, AFP, and tumor size were independent risk factors for prognosis. All independent risk factors were included in the nomogram to predict CSS at 1-, 3-, and 5-years in early middle age patients with hepatocellular carcinoma. In the training cohort and validation cohort, the C-index of the prediction model was 0.728 (95%CI: 0.716–0.740) and 0.733 (95%CI: 0.715–0.751), respectively. The calibration curve showed that the predicted value of the prediction model is highly consistent with the observed value. AUC also suggested that the model has good discrimination. DCA suggested that the nomogram had better predictive power than T staging.

**Conclusion:**

We constructed a new nomogram to predict CSS in middle-aged patients with early-stage hepatocellular carcinoma. This prediction model has good accuracy and reliability, which can help patients and doctors to judge prognosis and make clinical decisions.

## Introduction

Primary liver cancer is the sixth most commonly diagnosed cancer and the third leading cause of cancer-related death worldwide, with ~906,000 new cases and deaths in 2020, and hepatocellular carcinoma (HCC) accounts for the vast majority (75–85%) of primary liver cancers (1). Although some risk factors such as hepatitis B virus (HBV) and aflatoxin B1 (AFB1) are declining in many regions due to public health measures, the incidence of liver cancer is still rising, possibly because of the increasing prevalence of obesity and diabetes (2). Due to a variety of diagnostic methods, including ultrasonography, serum alpha-fetoprotein (AFP), computed tomography (CT), and magnetic resonance imaging (MRI) being widely used, a more significant proportion of early HCC patients are diagnosed (3). Besides, a majority of HCC patients are aged between 40 and 60. Hence, the survival prognosis of this group of patients with early HCC remains a significant concern.

Many risk factors are affecting the survival prognosis in patients with HCC, including age (4), race (5), histological grade (6), chemotherapy (7), surgery (8), tumor size (9), and so on. To the best of our knowledge, numerous nomogram prediction models based upon the above factors for HCC have been constructed (10–12), as well as prognostic nomogram for early-stage HCC (13). However, there have been no clinical studies and statistical tools that evaluated the predictive factors for early-stage HCC patients aged between 40 and 60. Considering the earlier the stage and the younger the patient, the better the prognosis and the more meaningful the prediction, we aim to extract data from the Surveillance, Epidemiology, and End Results (SEER) database to develop and validate a practical nomogram for predicting the cancer-specific survival (CSS) of patients with early HCC aged between 40 and 60 by integrating some significant variables so that clinicians can make better decisions.

At present, artificial intelligence has been widely used in human health care. Shubham et al. (14) used deep learning methods to identify glomerulus in human kidney tissue images. Movassagh et al. (15) used a new method to train neural networks. Mohan et al. (16) used machine learning to predict heart disease. Iwendi et al. (17, 18) used machine learning to classify COVID-19 patients and make health predictions. Kumar et al. (19) used neural networks to predict COVID-19. Ngabo et al. (20) use machine learning architectures to tackle urban pandemics. In this study, we used conventional nomogram to construct a new predictive model to predict the survival of middle-aged HCC patients. This prediction model can help HCC patients and doctors to develop clinical and follow-up strategies.

## Patients and Methods

### Data Source and Data Extraction

We downloaded clinicopathological information of all patients with HCC from 2010 to 2018 from the SEER database. We collected social and demographic information (age, sex, race, year of diagnosis, and marriage), tumor information (tumor stage, tumor profile, tumor size, and histological grade), and treatment information (radiation, chemotherapy, and surgery). Because SEER is a public database, patient information is not identifiable, so our study does not require ethical approval and patients' informed consent. Our analysis follows the data usage rules of the SEER database.

**Figure 1:**
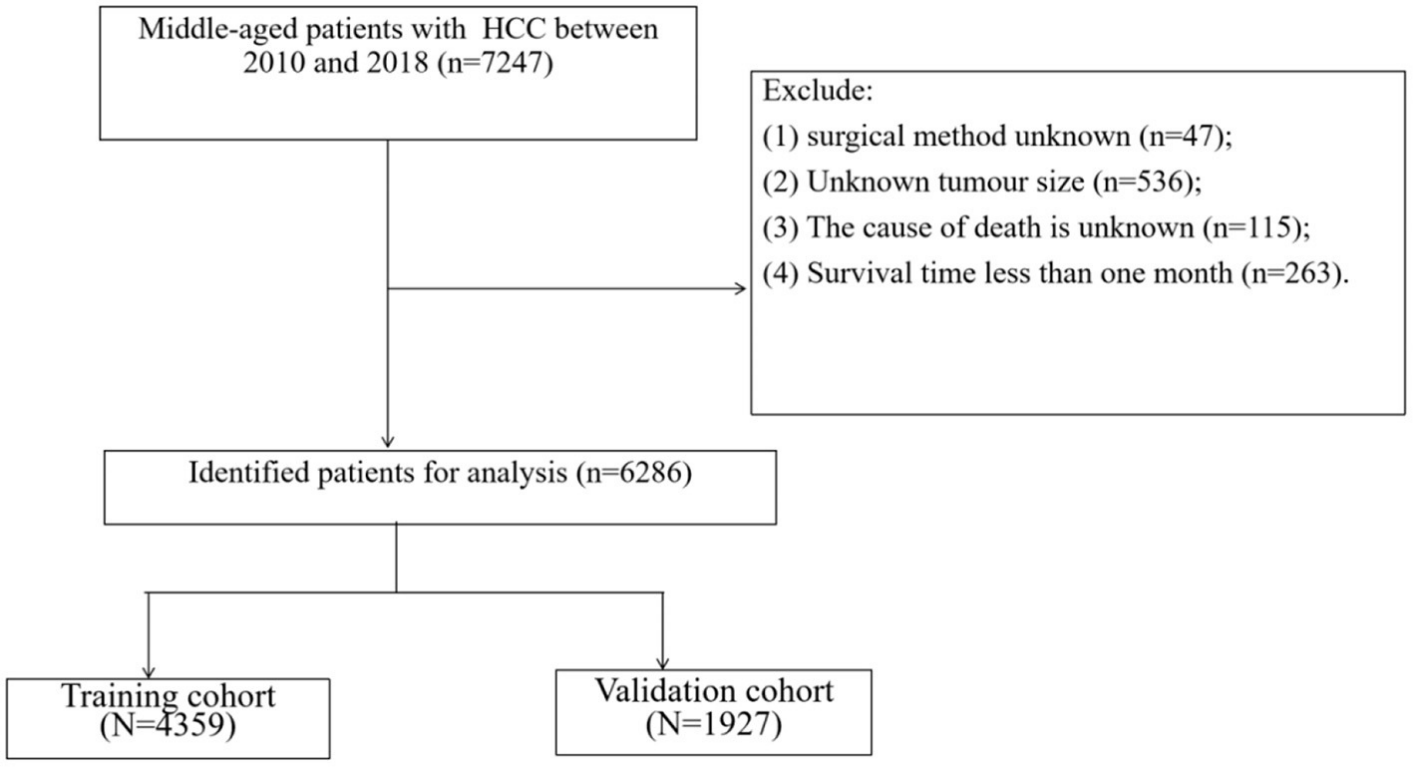
Flow chart of patient screening.

Inclusion criteria: (1) Pathological diagnosis of HCC; (2) Aged 40–60; (3) The years of diagnosis were 2010–2018. Exclusion criteria: (1) Surgical method unknown; (2) Unknown tumor size; (3) The cause of death is unknown; (4) Survival time <1 month. The patient inclusion and exclusion procedures are shown in Figure 1.

Patients were categorized as white, black, and others (American Indian/AK Native, Asian/Pacific Islander). The histological tumor grading of the patients was divided into grades I to IV, which were highly differentiated, moderately differentiated, poorly differentiated, and undifferentiated. The surgical methods of patients are divided into three categories: local tumor destruction, lobectomy, and transplant. AFP can be positive or negative.

### Construction and Validation of the Nomogram

All patients were randomly assigned to a training cohort (70%) or a validation cohort (30%). Univariate Cox regression analysis was used to identify prognostic risk factors in middle-aged patients with early-stage HCC in the training cohort. Multivariate Cox regression analysis analyzed the independent risk factors for prognosis. All independent risk factors were included in a nomogram to predict patients' CSS. A series of validation methods were used to validate the accuracy and discrimination of the prediction model, including Consistency index (C-index), calibration curve, area under the receiver operating curve (AUC).

### Clinical Utility

A decision analysis curve (DCA) is a method to evaluate the practical value of the model by calculating the net income under different thresholds. We used DCA to assess the clinical utility of the nomogram prediction model and compared it with conventional T staging. At the same time, we divided all patients into low-risk and high-risk groups based on each patient's nomogram score. Log-rank test and Kaplan-Meier (K-M) curve were used to compare survival differences among patients in different groups.

### Statistical Analysis

Counting data were expressed as frequency (%), and differences between groups were analyzed by Chi-square or non-parametric *U*-tests. Measurement data (age and tumor size) were described using mean and standard deviation, and differences between groups were analyzed using non-parametric *U*-tests. Univariate and multivariate Cox regression models examined the survival and prognostic factors of the patients. Log-rank test and Kaplan-Meier (K-M) curve were used to compare survival differences between groups. R software 4.1.0 and SPSS 26.0 were used for statistical analysis. A *P*-value < 0.05 was considered statistically significant.

## Results

### Clinical Features

A total of 6,286 patients with early-stage HCC aged between 40 and 60 were included in the study. The mean age of all patients was 54.4 ± 4.1 years, 4,298 (68.4%) patients were white, 5,005 (79.6%) patients were male, 2,799 (44.5%) patients were married, and 3,386 (53.9%) patients were AFP positive. The histological grades were 666 (10.6%) in grade I, 1,050 (16.7%) in grade II, 309 (4.92%) in grade III, and 16 (0.25%) in grade IV. There were 3,976 patients (63.3%) with stage T1 tumors, with a mean tumor size of 35.5 ± 32.2 mm. Partial tumor destruction was performed in 1,183 (18.8%) patients, lobectomy was performed in 702 (11.2%) patients, and liver transplantation was performed in 769 (12.2%) patients. Two thousand nine hundred and fourteen (46.4%) patients received chemotherapy, and 507 (8.07%) received radiotherapy. The clinicopathological information of all patients is shown in Table 1. There was no significant difference between the training cohort and the validation cohort.

**Table 1:** Clinicopathological characteristics of patients with HCC.

	**All**	**Training cohort**	**Validation cohort**	
	***N* = 6,286**	***N* = 4,359**	**2*N* = 1,927**	***p***
Age	54.4 (4.10)	54.4 (4.11)	54.4 (4.10)	0.810
**Race**				0.058
White	4,298 (68.4%)	2,940 (67.4%)	1,358 (70.5%)	
Black	1,004 (16.0%)	715 (16.4%)	289 (15.0%)	
Other	984 (15.7%)	704 (16.2%)	280 (14.5%)	
**Sex**				0.447
Male	5,005 (79.6%)	3,459 (79.4%)	1,546 (80.2%)	
Female	1,281 (20.4%)	900 (20.6%)	381 (19.8%)	
**Year of diagnosis**				0.762
2010–2014	3,928 (62.5%)	2,718 (62.4%)	1,210 (62.8%)	
2015–2018	2,358 (37.5%)	1,641 (37.6%)	717 (37.2%)	
**Marriage**				0.845
No	3,487 (55.5%)	2,414 (55.4%)	1,073 (55.7%)	
Married	2,799 (44.5%)	1,945 (44.6%)	854 (44.3%)	
**AFP**				0.853
Negative	1,848 (29.4%)	1,279 (29.3%)	569 (29.5%)	
Positive	3,386 (53.9%)	2,357 (54.1%)	1,029 (53.4%)	
Unknown	1,052 (16.7%)	723 (16.6%)	329 (17.1%)	
**Grade**				0.818
I	666 (10.6%)	453 (10.4%)	213 (11.1%)	
II	1,050 (16.7%)	725 (16.6%)	325 (16.9%)	
III	309 (4.92%)	211 (4.84%)	98 (5.09%)	
IV	16 (0.25%)	10 (0.23%)	6 (0.31%)	
Unknown	4,245 (67.5%)	2,960 (67.9%)	1,285 (66.7%)	
**T**				0.162
T1	3,976 (63.3%)	2,732 (62.7%)	1,244 (64.6%)	
T2	2,310 (36.7%)	1,627 (37.3%)	683 (35.4%)	
Tumor size	35.5 (32.2)	35.4 (32.0)	35.6 (32.7)	0.830
**Surgery**				0.325
No	3,632 (57.8%)	2,549 (58.5%)	1,083 (56.2%)	
Local tumor destruction	1,183 (18.8%)	816 (18.7%)	367 (19.0%)	
Lobectomy	702 (11.2%)	473 (10.9%)	229 (11.9%)	
Transplant	769 (12.2%)	521 (12.0%)	248 (12.9%)	
**Chemotherapy**				0.965
No/unknown	3,372 (53.6%)	2,337 (53.6%)	1,035 (53.7%)	
Yes	2,914 (46.4%)	2,022 (46.4%)	892 (46.3%)	
**Radiation**				0.370
No/unknown	5,779 (91.9%)	3,998 (91.7%)	1,781 (92.4%)	
Yes	507 (8.07%)	361 (8.28%)	146 (7.58%)	

### Univariate and Multivariate Cox Regression Analysis

In the training cohort, univariate Cox regression analysis suggested that sex, marriage, year of diagnosis, race, histological tumor grade, T stage, surgery, chemotherapy, AFP, and tumor size were correlated with prognosis. All related risk factors were included in multivariate Cox regression analysis. The results showed that sex, marriage, race, histological tumor grade, T stage, surgery, chemotherapy, AFP, and tumor size were independent risk factors for patient prognosis. The univariate and multivariate results are shown in Table 2.

**Table 2:** Univariate and multivariate analyses of CSS in training cohort.

	**Univariate**			**Multivariate**		
	**HR**	**95%CI**	***P***	**HR**	**95%CI**	***P***
Age	1.01	0.99–1.02	0.355			
**Race**						
White	Reference			Reference		
Black	1.16	1.03–1.31	0.017	1.035	0.913–1.173	0.589
Other	0.63	0.55–0.74	<0.001	0.669	0.575–0.777	<0.001
**Sex**						
Male	Reference			Reference		
Female	0.82	0.73–0.93	0.002	0.013	0.855–0.756	
**Year of diagnosis**						
2010–2014	Reference			Reference		
2015–2018	0.88	0.79–0.98	0.024	0.906	0.809–1.013	0.084
**Marriage**						
No	Reference			Reference		
Married	0.66	0.6–0.73	<0.001	0.786	0.712–0.869	<0.001
**AFP**						
Negative	Reference			Reference		
Positive	1.87	1.66–2.1	<0.001	1.661	1.475–1.872	<0.001
Unknown	1.25	1.07–1.47	0.006	1.225	1.044–1.438	0.013
**Grade**						
I	Reference			Reference		
II	1.14	0.92–1.42	0.231	1.348	1.079–1.684	0.009
III	2.29	1.77–2.96	<0.001	1.987	1.529–2.583	<0.001
IV	1.78	0.66–4.82	0.256	3.531	1.284–9.71	0.014
Unknown	2.08	1.74–2.5	<0.001	1.155	0.959–1.391	0.129
**T**						
T1	Reference			Reference		
T2	1.14	0.92–1.42	0.231	1.511	1.372–1.663	<0.001
Tumor size	1	1–1.001	<0.001	1.003	1.002–1.003	<0.001
**Surgery**						
No	Reference			Reference		
Local tumor destruction	0.48	0.42–0.55	<0.001	0.469	0.408–0.538	<0.001
Lobectomy	0.34	0.28–0.41	<0.001	0.279	0.225–0.347	<0.001
Transplant	0.08	0.06–0.11	<0.001	0.074	0.053–0.103	<0.001
**Chemotherapy**						
No/unknown	Reference			Reference		
Yes	1.36	1.16–1.61	<0.001	0.757	0.685–0.837	<0.001
**Radiation**						
No/unknown	Reference					
Yes	1.14	1.03–1.25	0.008			

### Construction and Validation of the Nomogram

**Figure 2:**
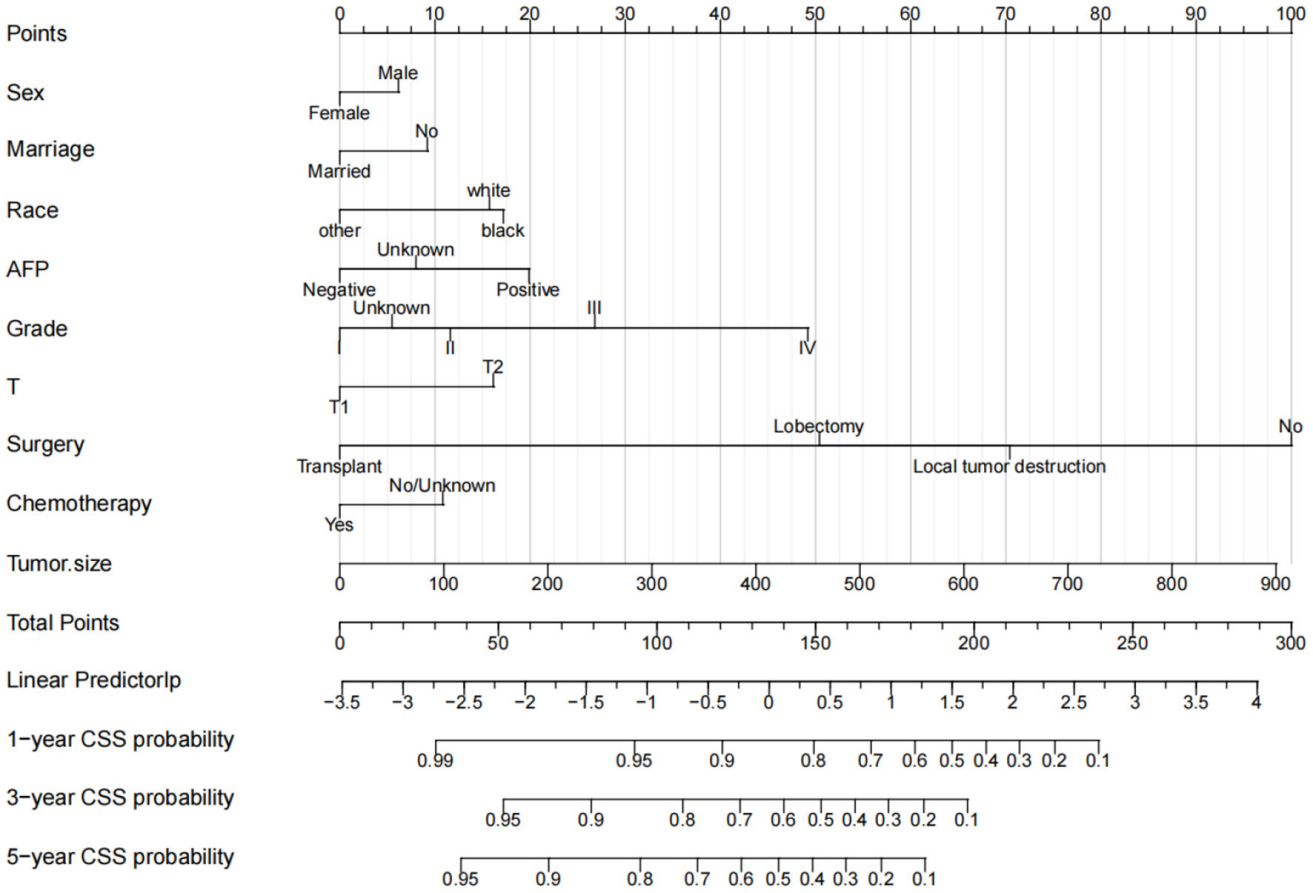
The nomogram of CSS in middle-aged patients with HCC at 1-, 3-, and 5-year.

**Figure 3:**
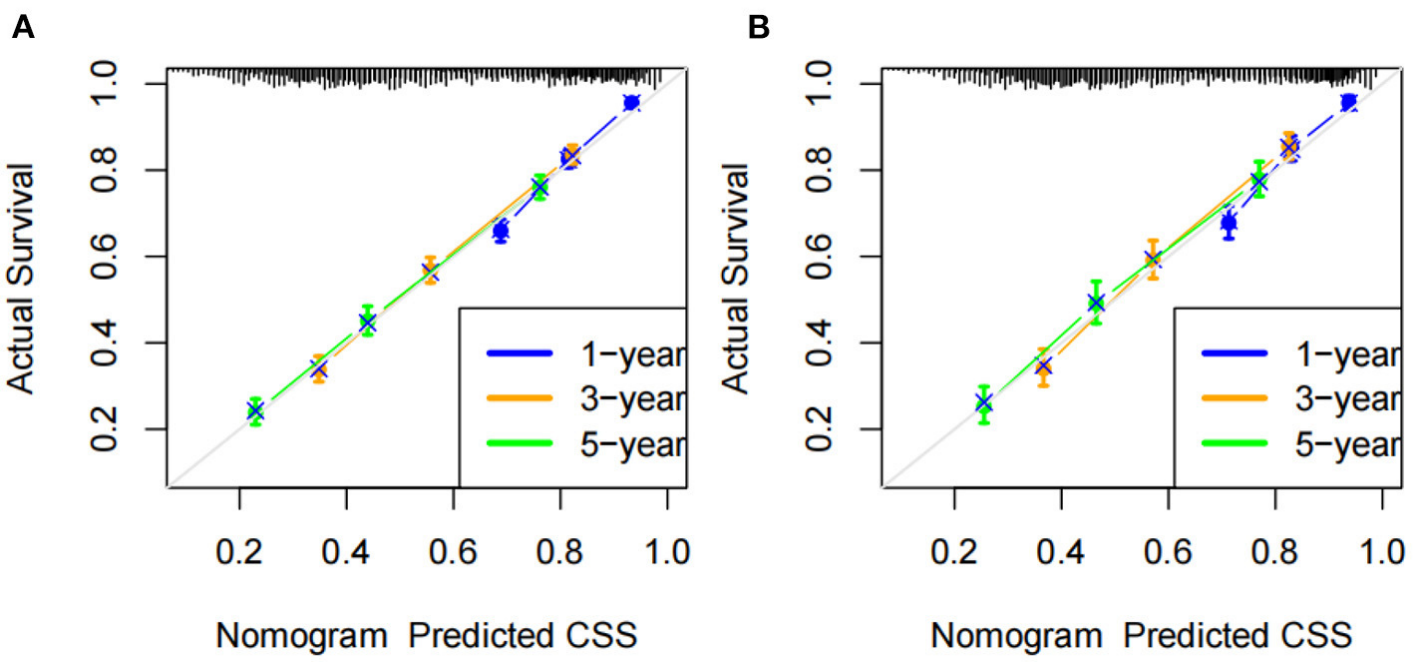
Calibration curve of the nomogram. **(A)** Calibration curves of 1-, 3-, and 5-year CSS in training cohort; **(B)** calibration curves of 1-, 3-, and 5-year CSS in the validation cohort.

**Figure 4:**
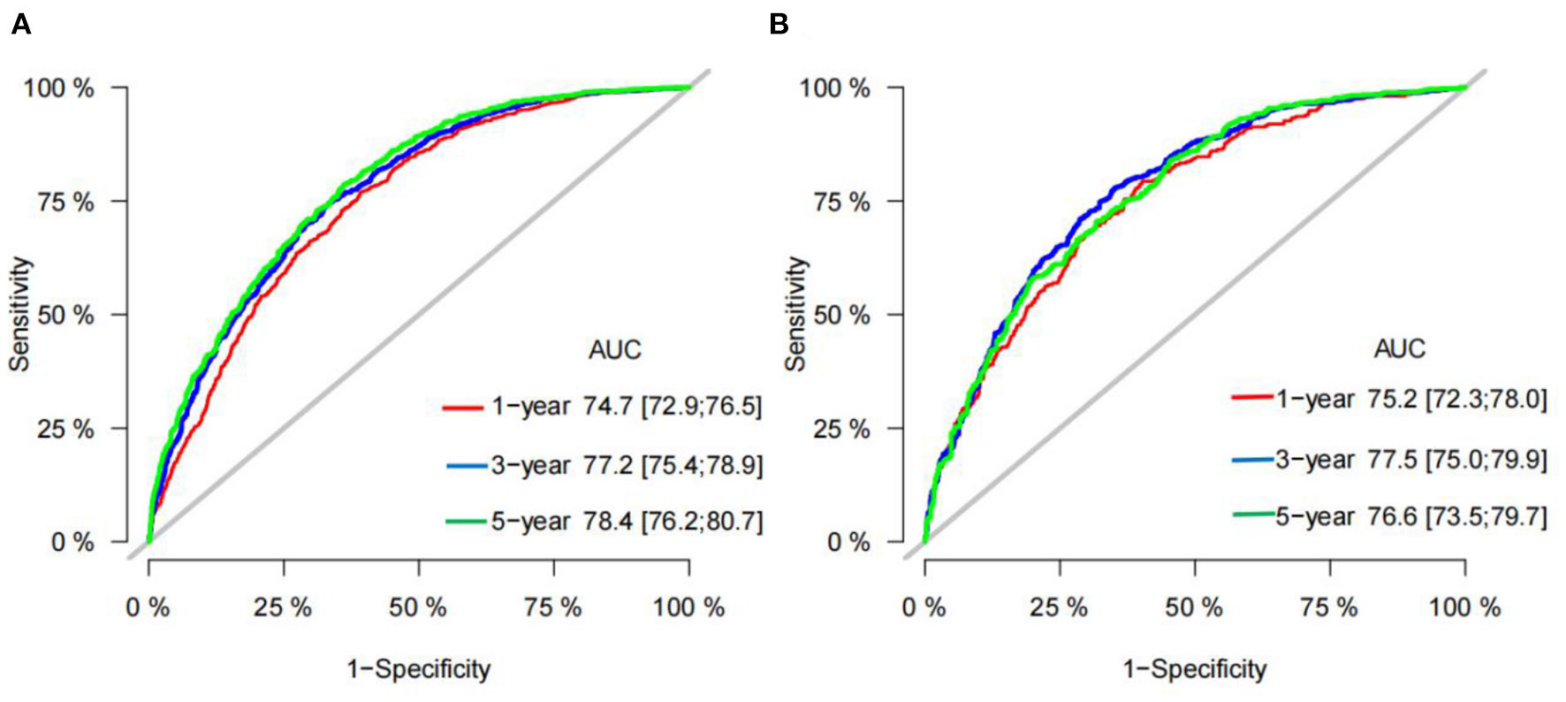
AUC for predicting 1-, 3-, and 5-year CSS in training cohort **(A)** and validation cohort **(B)**.

All independent risk factors were included in the nomogram to predict 1-, 3-, and 5-year CSS in early middle-aged HCC patients (Figure 2). Tumor size and operation were the most important factors influencing patient survival, followed by histological tumor grade, AFP, and T stage. In addition, race, marriage, and sex also influenced the prognosis of patients. In the training and validation cohort, the C-index of the prediction model was 0.728 (95%CI, 0.716–0.740) and 0.733 (95%CI, 0.715–0.751), respectively, indicating that the model had good discrimination. Calibration curves of the training cohort and validation cohort showed that the predicted value of the prediction model is highly consistent with the actual observed value (Figure 3), indicating that the model has good accuracy. In addition, the AUC of the training cohort and validation cohort also suggested that the model had good discrimination (Figure 4).

### Clinical Application of the Nomogram

**Figure 5:**
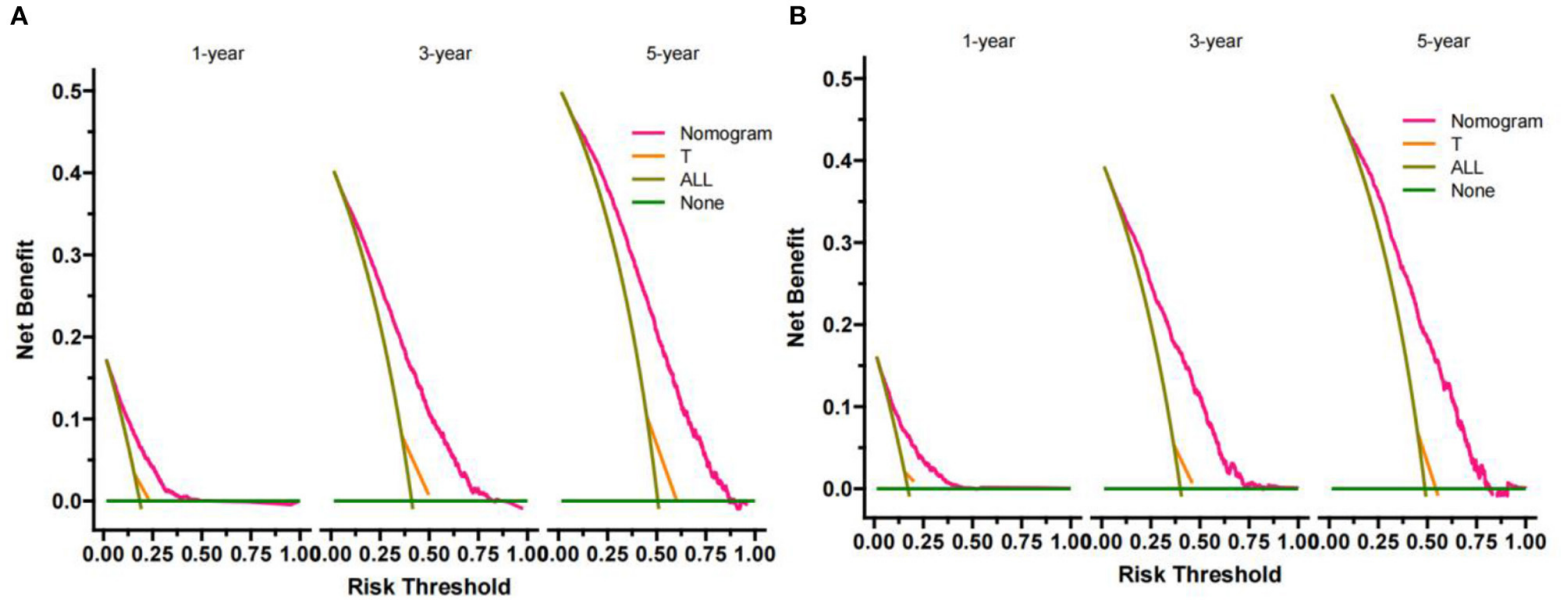
DCA of the nomogram in training cohort **(A)** and validation cohort **(B)**. The Y-axis represents a net benefit, and the X-axis represents threshold probability. The green line means no patients died, and the dark green line means all patients died. When the threshold probability is between 0 and 75%, the net benefit of the model exceeds all deaths or none.

**Figure 6:**
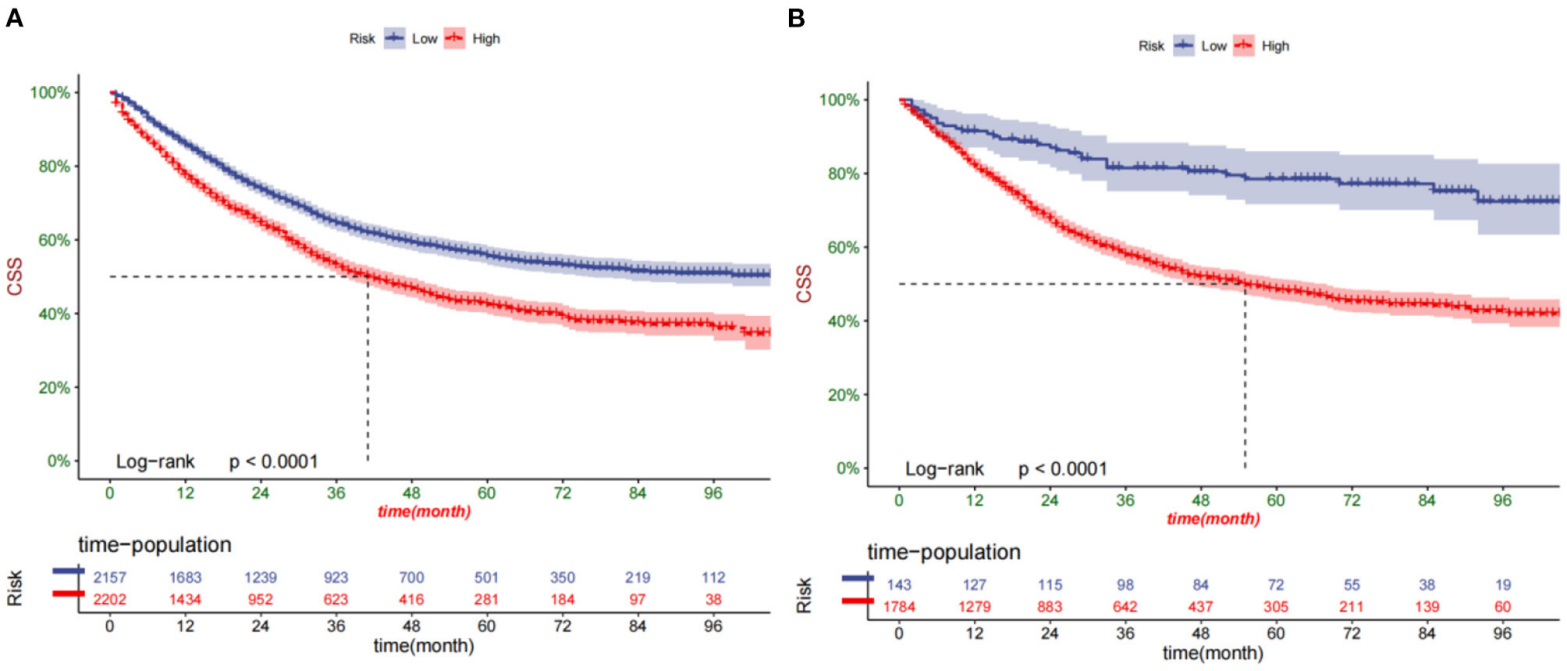
Kaplan-Meier curves of patients in the low-risk and high-risk groups in training cohort **(A)** and validation cohort **(B)**.

**Figure 7:**
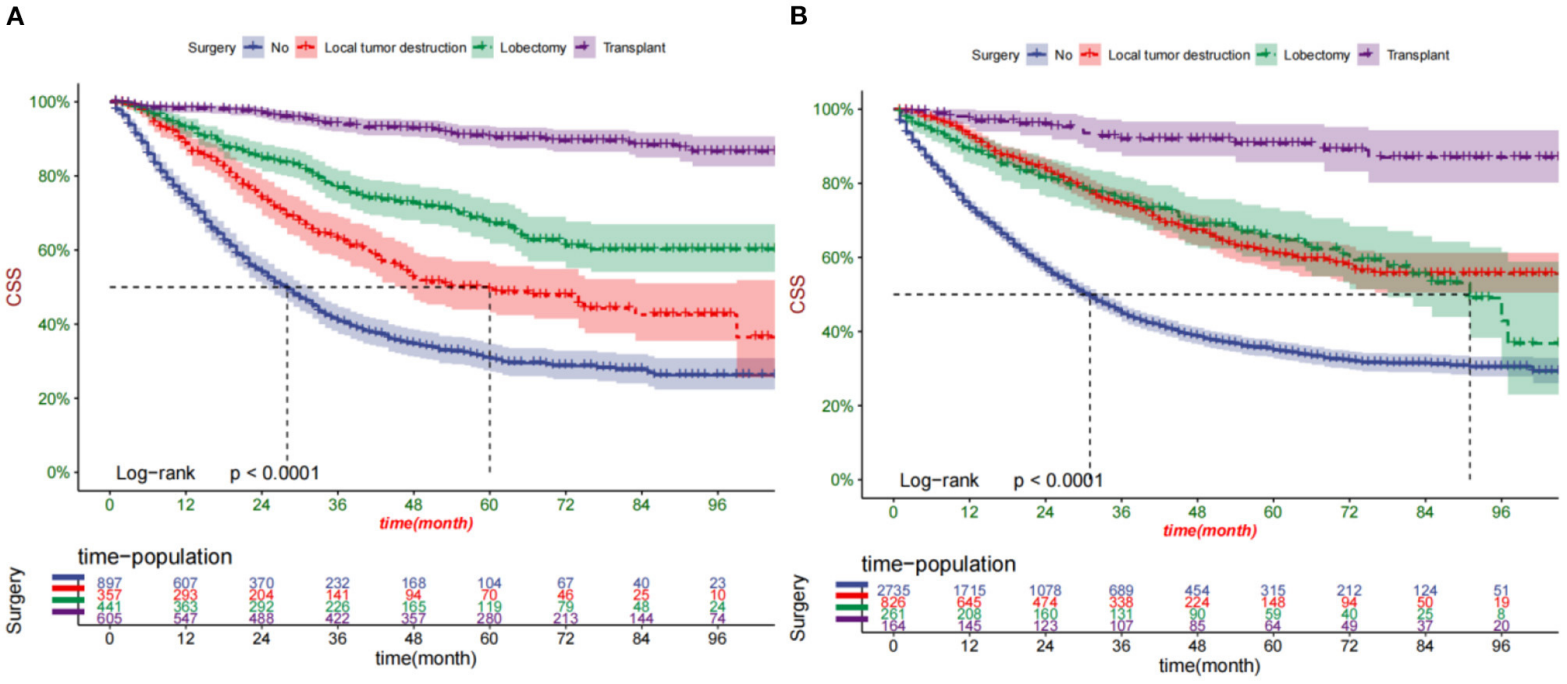
Kaplan-Meier curves of patients with different surgical procedures in the low-risk group **(A)** and high-risk group **(B)**.

DCA suggested that the nomogram had better predictive power than the T-staging (Figure 5). All patients were assigned to the high-risk group based on their nomogram scores (total >133.2) and low-risk group (total ≤ 133.2). In the training and validation cohort, patients in the low-risk group had significantly higher survival rates than those in the high-risk group (Figure 6). In the high-risk group, the 1-, 3-, and 5-year survival rates were 78.2, 53.5, and 43.3%, respectively. In the low-risk group, the 1-, 3-, and 5-year survival rates were 84.7, 67.9, and 59.8%, respectively. In the low-risk group, survival was highest among patients who received liver transplantation and lobectomy (Figure 7A). Survival of patients who received liver transplantation was highest in the high-risk group (Figure 7B).

## Discussion

Predictive models for cancer patients have been widely established, including nomograms for predicting survival in HCC patients. Yan et al. (13) constructed a nomogram to predict cancer-specific survival in early hepatocellular patients. Kong et al. (21) constructed a nomogram to predict survival in young patients with hepatocellular carcinoma. Hu et al. (22) developed a nomogram to predict survival in bone metastatic hepatocellular carcinoma patients. Liu et al. (23) constructed a nomogram to predict cancer-specific survival in all patients with hepatocellular carcinoma. Similarly, Ni et al. (24) constructed nomograms to predict survival in all hepatocellular patients. Nevertheless, prognosis and risk of death in HCC patients vary greatly with age. Middle-aged people have a high incidence of hepatocellular carcinoma, and their prognostic survival factors are different from those of other age groups. For example, marriage and race can affect the survival of patients, so a special nomogram is needed to predict patient survival.

Our study extracted information on early-stage HCC patients aged between 40 and 60 from the SEER database. We split the entire cohort into training and validation groups. Differences between the two groups regarding various essential variables such as marriage status, AFP level, grade, stage, and treatments were tested. All tests showed *p*-values > 0.05, indicating no significant difference between the training and validation groups. Univariate and multivariate Cox analysis found that numerous factors significantly affected CSS, including sex, race, marital status, AFP level, grade, T stage, tumor size, surgery, and chemotherapy. At the same time, other variables such as age and year of diagnosis were not identified as prognostic significance. That is partly because the population we selected is strictly defined, and there was so little difference between patients about age and year of diagnosis.

Social and demographic variables (age, race, and marital status) were identified as prognosticators of HCC. In our study, marital status is a relatively important factor for survival prediction, parallel to many previous studies (13, 25). This is partly because most single, separated, and divorced cancer patients experience more stress and pain than married patients (26). Besides, married patients have better adherence to prescribed treatments than unmarried patients, leading to better cancer control (27). Furthermore, several biological, psychological, and social theories can also explain this phenomenon. A systematic review and meta-analysis indicated significant racial disparities in HCC prognosis in the United States, with Black patients having worse overall survival and Hispanic and Asian patients having better overall survival than White patients (28). Nikita Sandeep Wagle et al. found that Black patients receive curative treatment more minor than White because of social and economic situations (29), which may partly explain this phenomenon. Consistently, we found Black patients having worse CSS, closely followed by the White (*P* = 0.589), and other races having better CSS (*P* < 0.001; Table 2).

As for the tumor features (pathological grade, T stage, and tumor size) variables included in the nomogram, the tumor size has the most significant effect for CSS. A retrospective study suggests that tumor size is a vital independent prognostic factor in T2 solitary HCC after curative liver resection (30). In addition, the Milan criteria of liver transplantation strictly limited tumor size, which means that tumors with smaller diameters can be treated in more ways and get better treatment (31). Our results consistently reflected the relationship between tumor size and CSS and quantified its impact on CSS of a specified population compared with previous studies.

There are several kinds of treatment methods for patients with early-stage HCC, including liver resection (LR), ablation, and orthotopic liver transplantation (OLT) (32). Liver transplantation is considered an ideal treatment for selected patients with HCC, as it removes both the tumor and potential cirrhosis, with a 5-year survival rate exceeding 70% (33). Our results once again confirmed its superiority in the treatment of early-stage HCC, and its score is the lowest in the nomogram, which indicated the best outcome. However, the shortage of donor organs has severely limited the utilization of liver transplantation (34). In our results, lobectomy is inferior to liver transplantation but superior to local tumor destruction such as ablation. A systematic review of randomized controlled trials with Meta-analysis and sequential trial analysis found no statistical difference between radiofrequency ablation (RFA) and hepatic resection (HR) in overall survival at 1 and 3 years. Still, RFA is resulted in decreased overall survival compared with HR at 5 years (35). A retrospective study showed that lobectomy has better overall survival and higher cancer-specific survival than thermal ablation (36). Moreover, chemotherapy such as sorafenib and lenvatinib is also an effective treatment for HCC (32). Therefore, it is suggested that surgery (OLT, LR, or RFA) combined with chemotherapy is an ideal choice for early-stage HCC patients aged between 40 and 60.

In our prediction model, the AUC of 1-, 3-, 5-year CSS is 0.747 (95%CI: 72.9–76.5), 77.2 (95%CI: 75.4–78.9), and 78.4 (95%CI: 76.2–80.7), respectively, in the training cohort. In the validation cohort, the AUC of 1-, 3-, 5-year CSS is 0.752 (95%CI: 72.3–78.0), 77.5 (95%CI: 75.0–79.9), and 76.6 (95%CI: 73.5–79.7), respectively (Figure 4). It indicates that our nomogram has relatively high accuracy in predicting CSS of early-stage HCC patients aged between 40 and 60. In addition, we also drew DCA curves to assess the clinical practicability of the nomogram, which suggested that the prediction model has better clinical application value than the traditional TNM staging system and can more accurately predict the 1-, 3-, 5-year CSS of early-stage HCC patients aged between 40 and 60.

However, our study has limitations. First of all, the nomogram was constructed based on the SEER database. It neither necessarily represents the rest of the world nor includes important factors such as alcohol intake, viral infection, cirrhosis, and liver function. Besides, our study was performed by retrospective analysis, which inevitably has a selection bias that is difficult to adjust. Moreover, our prediction model has only been validated internally, and external validation or further prospective studies are necessary. It is suggested that a multicenter study including more liver function variables be conducted.

## Conclusion

We explored cancer-specific survival factors in early middle-aged patients with HCC. We found that sex, marriage, race, histological tumor grade, T stage, surgery, chemotherapy, AFP, and tumor size were independent risk factors for patient prognosis. We constructed a new nomogram to predict CSS in patients with early-stage HCC in middle age using these risk factors. This prediction model has good accuracy and reliability, which can help patients and doctors to judge prognosis and clinical decisions.

## Data Availability Statement

Publicly available datasets were analyzed in this study. This data can be found here: https://seer.Cancer.gov/.

## Author Contributions

JT and CW designed the study, revised the article critically, reviewed, and edited the article. CW, JT, and HL collected and analyzed the data. CW drafted the initial manuscript. All authors approved the final manuscript.

## Funding

This work was supported by a grant from the Science and Technology Department of Sichuan Province, China (Joint Research Fund, No. 2019YFH0056).

## Conflict of Interest

The authors declare that the research was conducted in the absence of any commercial or financial relationships that could be construed as a potential conflict of interest.

## Publisher's Note

All claims expressed in this article are solely those of the authors and do not necessarily represent those of their affiliated organizations, or those of the publisher, the editors and the reviewers. Any product that may be evaluated in this article, or claim that may be made by its manufacturer, is not guaranteed or endorsed by the publisher.
